# Metformin Ameliorates Hepatic Steatosis and Inflammation without Altering Adipose Phenotype in Diet-Induced Obesity

**DOI:** 10.1371/journal.pone.0091111

**Published:** 2014-03-17

**Authors:** Shih-Lung Woo, Hang Xu, Honggui Li, Yan Zhao, Xiang Hu, Jiajia Zhao, Xin Guo, Ting Guo, Rachel Botchlett, Ting Qi, Ya Pei, Juan Zheng, Yiming Xu, Xiaofei An, Lulu Chen, Lili Chen, Qifu Li, Xiaoqiu Xiao, Yuqing Huo, Chaodong Wu

**Affiliations:** 1 Department of Nutrition and Food Science, Texas A&M University, College Station, Texas, United States of America; 2 Department of Endocrinology, Union Hospital, Tongji College of Medicine, Huazhong University of Science and Technology, Wuhan, Hubei, China; 3 Department of Stomatology, Union Hospital, Tongji College of Medicine, Huazhong University of Science and Technology, Wuhan, Hubei, China; 4 Vascular Biology Center, Department of Cellular Biology and Anatomy, Medical College of Georgia, Georgia Regents University, Augusta, Georgia, United States of America; 5 Drug Discovery Center, Key Laboratory of Chemical Genomics, Peking University Shenzhen Graduate School, Shenzhen, China; 6 Department of Endocrinology, the First Affiliated Hospital of Chongqing Medical University, Chongqing, China; 7 The Laboratory of Lipid & Glucose Metabolism, the First Affiliated Hospital of Chongqing Medical University, Chongqing, China; Pennington Biomedical Research Center, United States of America

## Abstract

Non-alcoholic fatty liver disease (NAFLD) is closely associated with obesity and insulin resistance. To better understand the pathophysiology of obesity-associated NAFLD, the present study examined the involvement of liver and adipose tissues in metformin actions on reducing hepatic steatosis and inflammation during obesity. C57BL/6J mice were fed a high-fat diet (HFD) for 12 weeks to induce obesity-associated NAFLD and treated with metformin (150 mg/kg/d) orally for the last four weeks of HFD feeding. Compared with HFD-fed control mice, metformin-treated mice showed improvement in both glucose tolerance and insulin sensitivity. Also, metformin treatment caused a significant decrease in liver weight, but not adiposity. As indicated by histological changes, metformin treatment decreased hepatic steatosis, but not the size of adipocytes. In addition, metformin treatment caused an increase in the phosphorylation of liver AMP-activated protein kinase (AMPK), which was accompanied by an increase in the phosphorylation of liver acetyl-CoA carboxylase and decreases in the phosphorylation of liver c-Jun N-terminal kinase 1 (JNK1) and in the mRNA levels of lipogenic enzymes and proinflammatory cytokines. However, metformin treatment did not significantly alter adipose tissue AMPK phosphorylation and inflammatory responses. In cultured hepatocytes, metformin treatment increased AMPK phosphorylation and decreased fat deposition and inflammatory responses. Additionally, in bone marrow-derived macrophages, metformin treatment partially blunted the effects of lipopolysaccharide on inducing the phosphorylation of JNK1 and nuclear factor kappa B (NF-κB) p65 and on increasing the mRNA levels of proinflammatory cytokines. Taken together, these results suggest that metformin protects against obesity-associated NAFLD largely through direct effects on decreasing hepatocyte fat deposition and on inhibiting inflammatory responses in both hepatocytes and macrophages.

## Introduction

Non-alcoholic fatty liver disease (NAFLD) is defined by fat deposition in hepatocytes (hepatic steatosis). In generally accepted concepts, NAFLD is comprised of simple steatosis, which may be benign, and non-alcoholic steatohepatitis (NASH), which is the advanced form of NAFLD. Simple steatosis progresses to NASH when the liver develops overt inflammation and necrotic damage that are not associated with alcohol consumption. It is now recognized that NASH is a leading causal factor of cirrhosis and hepatocellular carcinoma [1], [2]. Additionally, hepatic steatosis is a major contributor of dyslipidemia that works with or without insulin resistance to significantly increase the incidence of atherogenic cardiovascular diseases [3]. Given this, a better understanding of how to reduce hepatic steatosis and how to decrease liver inflammation are of critical importance in effectively managing NAFLD and fatty liver-associated metabolic and inflammatory diseases.

Because NAFLD is highly prevalent in obese populations [4], obesity-associated insulin resistance is considered as a factor that critically contributes to the development of NAFLD. Mechanistically, insulin resistance at both hepatic and systemic levels, along with hyperinsulinemia, acts to increase the expression of genes for lipogenic enzymes such as acetyl-CoA carboxylase 1 (ACC1) and fatty acid synthase (FAS) [5], [6] and to decrease the expression of genes for fatty acid oxidation including carnitine palmitoyltransferase 1a (CPT1a) [7]. These changes, in turn, bring about hepatic steatosis. As a primary “hit”, fat deposition is sufficient to trigger the inflammatory responses as indicated by the results from cultured hepatocytes [8], [9]. As another key characteristic of obesity, adipose tissue dysfunction has also been implicated in the development of NAFLD. Indeed, this role of dysfunctional adipose tissue is highlighted by the “second hit” hypothesis. In support of this, adipocyte-specific overexpression of monocyte chemoattractant protein-1 (MCP1), an inflammatory molecule up-regulated in adipose tissue of obese mice and human subjects, mediates the effect of adipose tissue inflammation to bring about an increase in hepatic triglyceride content [10]. These results and many others suggest that dysfunctional adipose tissue contributes to hepatic steatosis by increasing the delivery of fatty acid flux to the liver [2] and by impairing liver insulin signaling through adipose tissue-driven inflammation [11], [12]. Currently, a number of approaches that are capable of improving insulin sensitivity and adipose tissue functions, i.e., weight loss, metformin treatment, and insulin sensitization by thiazolidinediones (TZDs), have been considered for managing NAFLD [1], [13]–[15].

Metformin is a widely used anti-diabetic medicine that effectively lowers plasma glucose levels primarily by decreasing hepatic glucose production (HGP) and by improving lipid metabolism in both liver and muscle tissues [16]–[19]. At the cellular level, metformin activates AMP-activated protein kinase (AMPK). This serves as a key mechanism by which metformin treatment brings about a wide range of metabolic benefits [20]. Recent evidence also suggests that metformin is capable of inhibiting hepatic gluconeogenesis, a key flux whose increase contributes to elevation of HGP and hyperglycemia, through pathway(s) other than AMPK [21], [22]. Although the mechanisms underlying metformin actions are more complicated than what were thought before, there is accumulating evidence that demonstrates the beneficial effects of metformin treatment on improving hepatic steatosis and on inhibiting liver inflammation [13], [23]. At the systemic level, however, it remains to be a critical need to explore the extent to which the anti-NAFLD effects of metformin are related to alternations in both the liver and adipose tissue. Accordingly, it was hypothesized that metformin protects against NAFLD through direct effects on liver metabolic and inflammatory responses and through an indirect effect on improving adipose tissue phenotype. The results of the present study support that metformin acts directly to improve hepatocyte fat metabolism and to suppress inflammatory responses of both hepatocytes and macrophages. Interestingly, the protective role of metformin in reducing obesity-associated hepatic steatosis and inflammation appears to be independent of alterations in adipose tissue phenotype.

## Results

### Metformin treatment ameliorates HFD-induced systemic insulin resistance and glucose intolerance

**Figure 1:**
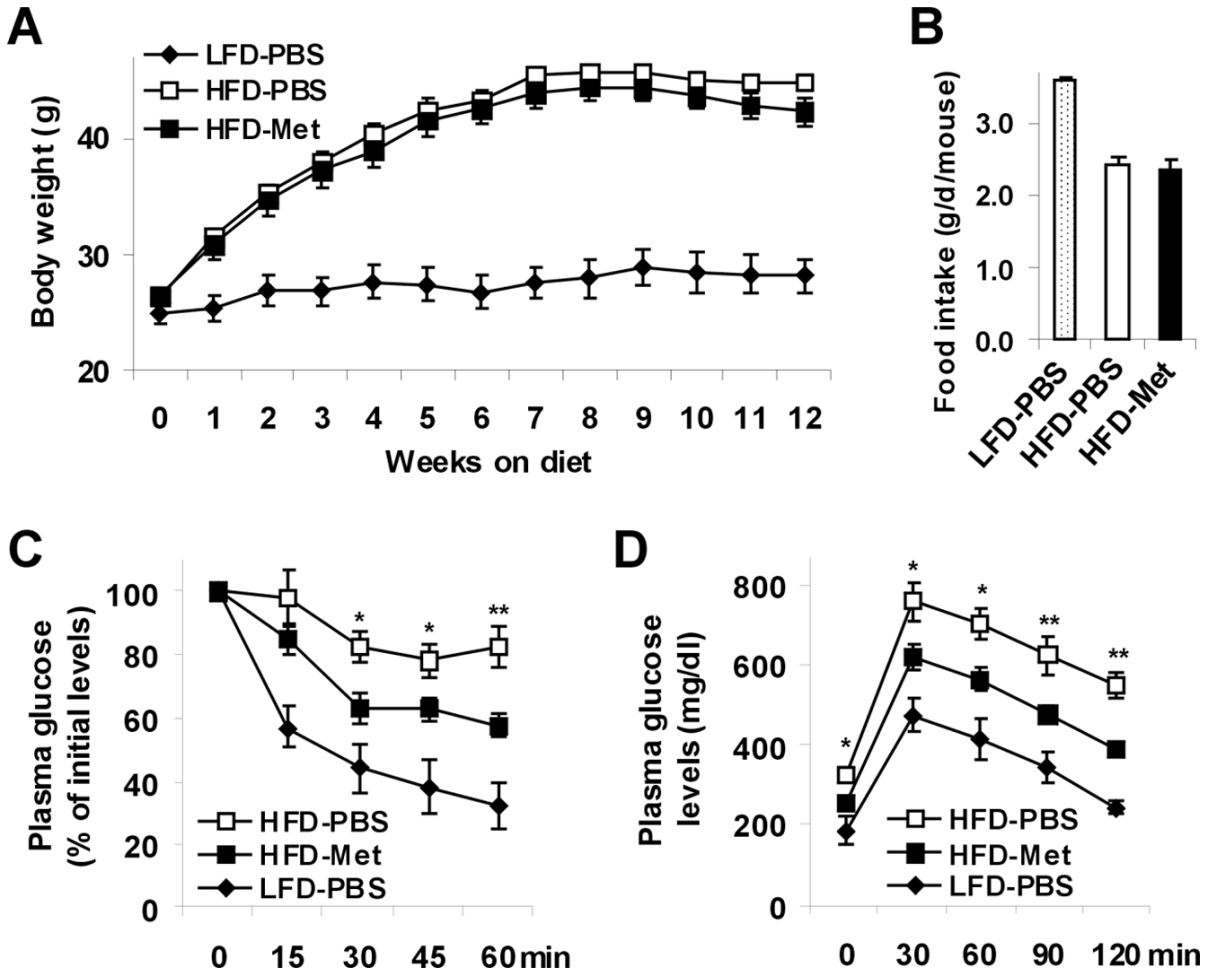
Metformin treatment ameliorates HFD-induced insulin resistance and glucose intolerance. Male C57BL/6J mice, at 5–6 weeks of age, were fed a high-fat diet (HFD) and treated with metformin (Met, 150 mg/kg body weight/d, in phosphate-buffered saline (PBS)) or PBS for the last 4 weeks of HFD feeding. As an additional control, gender- and age-matched mice were fed a low-fat diet (LFD) for 12 weeks and treated only with PBS for the last 4 weeks. Data are means ± SE, n = 6–10. (A) Body weight was monitored weekly during the feeding/treatment regimen. (B) Food intake was calculated based on food consumption per day per mouse. (C) Insulin tolerance tests (ITT). (D) Glucose tolerance tests (GTT). For C and D, mice were fasted for 4 hr and received an intraperitoneal injection of insulin (1 U/kg body weight) (C) or glucose (2 g/kg body weight) (D). *, *P*<0.05 and **, *P*<0.01 HFD-Met vs. HFD-PBS for the same time point (C and D).

**Figure 2:**
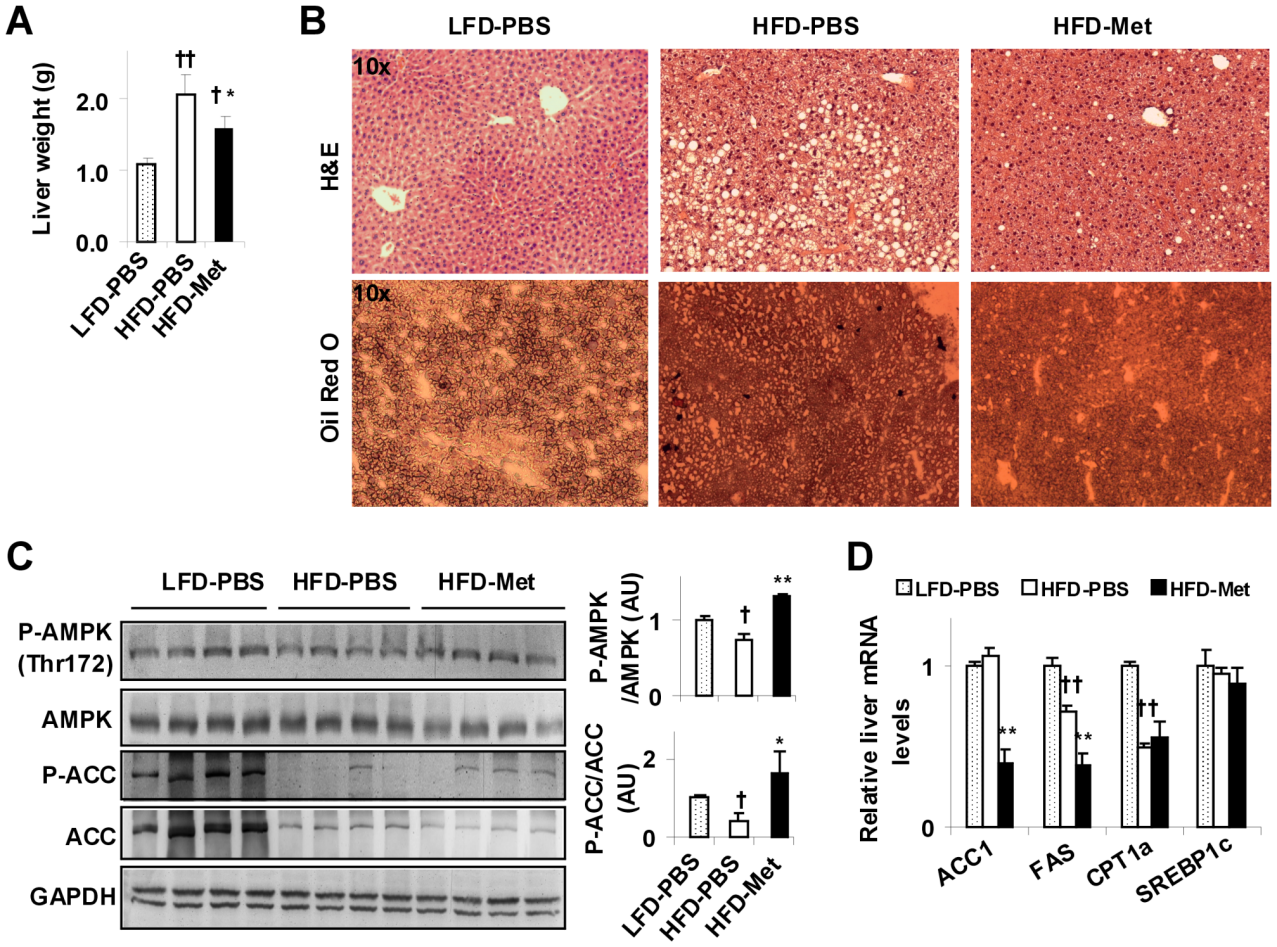
Metformin treatment ameliorates HFD-induced hepatic steatosis and increases liver AMPK phosphorylation. Mice were treated as described in Figure 1. After the feeding/treatment regimen, mice were fasted for 4 hr prior to collection of tissue samples. (A) Liver weight (n = 6–10). (B) Liver histology. Top panels, H&E staining; bottom panels, Oil-Red-O staining. (C) Liver AMPK signaling. Liver extracts were subjected to Western blot analyses. Ratios of phosphorylated AMPK to total AMPK (P-AMPK/AMPK) and phosphorylated ACC to total ACC (P-ACC/ACC) were quantified using densitometry and normalized to GAPDH (AU, arbitrary unit). (D) Liver mRNA levels of key lipid metabolic enzymes (genes) were analyzed using real-time PCR. For bar graphs (A, C, and D), data are means ± SE, n = 6–8. ^†^, *P*<0.05 and ^††^, *P*<0.01 HFD-PBS or HFD-Met vs. LFD-PBS (A, C, and D); *, *P*<0.05 and **, *P*<0.01 HFD-Met vs. HFD-PBS (A, C, and D).

**Figure 3:**
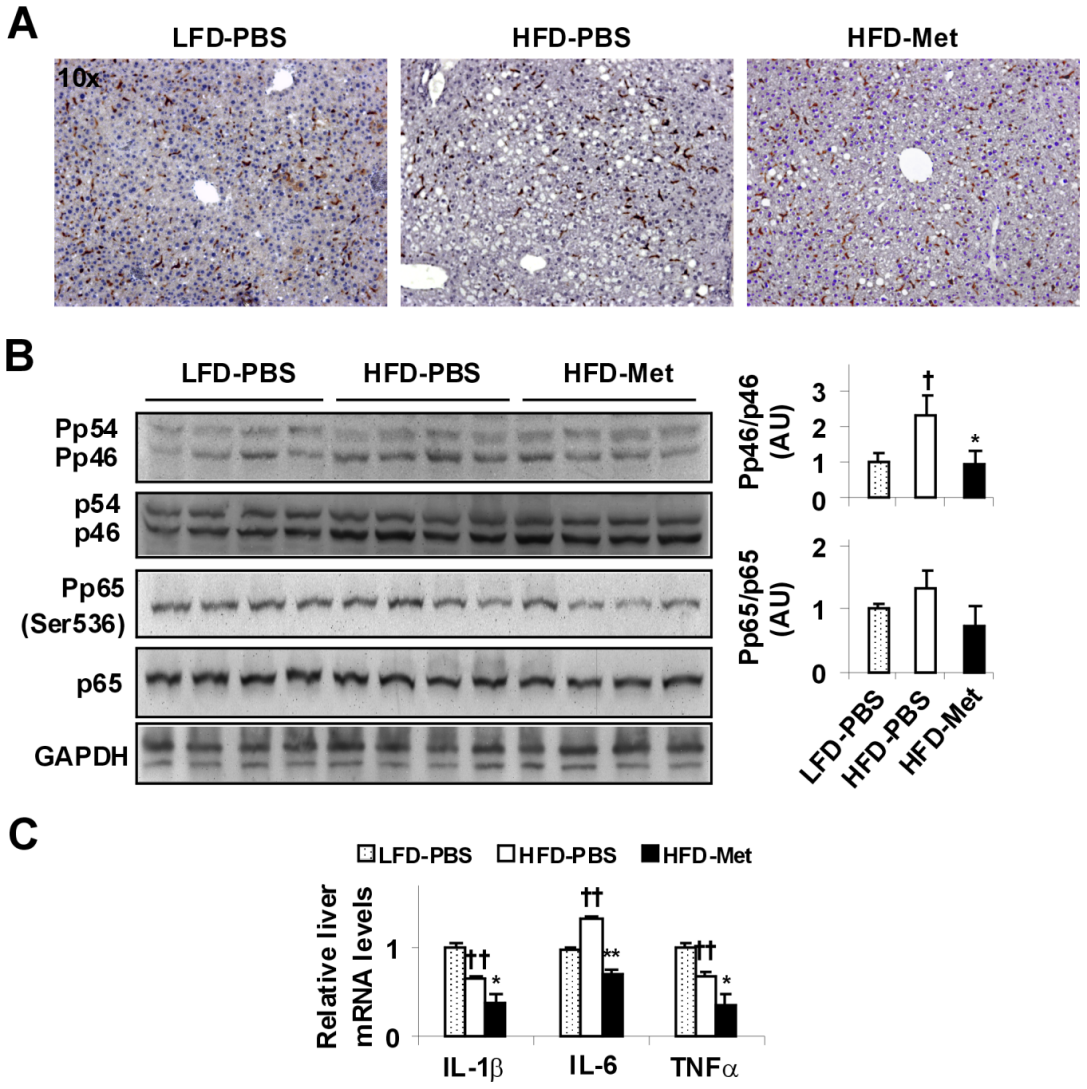
Metformin treatment ameliorates HFD-induced liver inflammatory responses. Mice were treated as described in Figure 1. After the feeding/treatment regimen, mice were fasted for 4 hr prior to collection of tissue samples. (A) Liver sections were stained for F4/80^+^ cells. (B) Liver inflammatory signaling. Liver extracts were subjected to Western blot analyses. Ratios of phosphorylated JNK1 to total JNK1 (Pp46/p46) and phosphorylated NF-κB p65 to total p65 (Pp65/p65) were quantified using densitometry and normalized to GAPDH. (C) Liver mRNA levels of proinflammatory cytokines were analyzed using real-time PCR. For bar graphs (B and C), data are means ± SE, n = 6–8. ^†^, *P*<0.05 and ^††^, *P*<0.01 HFD-PBS vs. LFD-PBS; *, *P*<0.05 and **, *P*<0.01 HFD-Met vs. HFD-PBS.

C57BL/6J mice were fed a high-fat diet (HFD) for 12 weeks to induce obesity (diet-induced obesity, DIO). Compared with age- and gender-matched mice that were fed a low-fat diet (LFD), HFD-fed and phosphate-buffered saline (PBS)-treated mice exhibited a marked increase in body weight (Figure 1A), as well as overt hepatic steatosis and increased liver inflammatory responses (see below, Figures 2 and 3). These results demonstrate the establishment of NAFLD in obese mice. In addition, HFD-fed and PBS-treated mice displayed a significant increase in the severity of insulin resistance and glucose intolerance (Figure 1, C and D) compared with LFD-fed and PBS-treated mice. Upon treatment with metformin, body weight of HFD-fed mice remained the same compared with HFD-fed and PBS-treated mice. Also, metformin treatment did not alter food intake of HFD-fed mice (Figure 1B, HFD-Met vs. HFD-PBS). However, metformin-treated mice showed a significant decrease in the severity of HFD-induced insulin resistance and glucose intolerance (Figure 1, C and D), which were indicated by changes in plasma levels of glucose in response to a peritoneal injection of insulin and glucose, respectively. Thus, treatment with metformin ameliorates HFD-induced systemic insulin resistance and glucose intolerance without altering body weight in DIO mice.

### Metformin treatment decreases HFD-induced hepatic steatosis and increases liver AMPK phosphorylation

NAFLD is commonly associated with obesity. Consistent with increased body weight, liver weight of HFD-fed and PBS-treated mice was much greater than that of LFD-fed mice (Figure 2A). Additionally, HFD-fed and PBS-treated mice displayed severe hepatic steatosis indicated by changes in liver sections stained with hematoxylin and eosin (H&E) and/or Oil-Red-O (Figure 2B). Given this, HFD-fed mice, serving as a mouse model of NAFLD, were used to examine the therapeutic effects of metformin, as well as the underlying mechanisms. Compared with HFD-fed and PBS-treated mice, HFD-fed and metformin-treated mice exhibited a significant decrease in liver weight (Figure 2A), which was accompanied by a marked decrease in the severity of hepatic steatosis (Figure 2B). Thus, treatment with metformin effectively ameliorates hepatic steatosis in obese mice. We next examined changes in liver AMPK phosphorylation, which may underlie the beneficial effects of metformin. Compared with LFD-fed and PBS-treated mice, HFD-fed and PBS-treated mice showed a significant decrease in liver AMPK phosphorylation (Figure 2C). This decrease was reversed by treatment with metformin as this was supported by the finding that liver AMPK phosphorylation in HFD-fed and metformin-treated mice was much greater than that in HFD-fed and PBS-treated mice (Figure 2C). Consistently, the phosphorylation of ACC, a substrate enzyme of AMPK, was decreased in HFD-fed and PBS-treated mice compared with that in LFD-fed mice. Upon treatment with metformin, liver ACC phosphorylation in HFD-fed mice was significantly increased compared with that of HFD-fed and PBS-treated mice (Figure 2C). We also examined changes in the mRNA levels of key enzymes that critically control the development of hepatic steatosis from livers of HFD-fed mice. In terms of regulating hepatic gene expression, the effects of HFD feeding have been previously studied in rodents by a number of investigators. However, HFD feeding increases liver FAS mRNA levels when compared with a chow diet [24], [25] and decreases liver FAS mRNA levels when compared with LFD feeding [26], [27]. Additionally, HFD feeding appears to have limited effects on liver expression of ACC and sterol regulatory element-binding protein 1c (SREBP1c), the latter is a transcription factor whose active form stimulates hepatic expression of lipogenic enzymes including ACC and FAS. In the represent study, we confirmed that HFD feeding decreased liver mRNA levels of FAS compared with LFD feeding. Meanwhile, HFD feeding did not significantly alter the mRNA levels of ACC and SREPBP1c. However, within all HFD-fed mice, treatment with metformin caused a significant decrease in the mRNA levels of ACC1 and FAS compared with PBS (Figure 2D). These results, together with decreased phosphorylation of liver ACC in metformin-treated mice, suggest a likely decrease in hepatic lipogenesis, which is consistent with the outcome of increased hepatic AMPK phosphorylation. Compared with LFD-fed and PBS-treated mice, HFD-fed and PBS-treated mice also displayed a decrease in liver mRNA levels of CPT1a, a rate-determining enzyme that transfers long-chain fatty acids into mitochondria for oxidation. Within HFD-fed mice, treatment with metformin tended to increase liver mRNA levels of CPT1a, but the increase was not statistically significant. Collectively, these results suggest that metformin ameliorates HFD-induced hepatic steatosis, and this effect of metformin is associated with an increase in liver AMPK phosphorylation.

### Metformin treatment inhibits HFD-induced liver inflammatory responses

Inflammation is the key factor that drives the progression of simple steatosis to NASH. We examined the content of macrophages/Kupffer cells (F4/80^+^ cells) in livers of the mice. Unlike adipose tissue which displays a marked increase in macrophage infiltration in response to HFD feeding as established by many publications, livers of HFD-fed and PBS-treated mice contained fewer numbers of F4/80^+^ cells (Figure 3A, quantitative data not shown). Also, treatment with metformin tended to increase liver content of F4/80^+^ cells. These results suggest that liver content of F4/80^+^ cells is not an ideal indicator of liver inflammatory responses. We then examined liver inflammatory signaling through JNK and NF-κB p65 and quantified the mRNA levels of proinflammatory cytokines to assess liver inflammatory responses. Compared with that in livers of LFD-fed and PBS-treated mice, the phosphorylation of JNK1 (p46) in livers of HFD-fed and PBS-treated mice was significantly increased (Figure 3B). A similar trend was also observed in the phosphorylation of NF-κB p65 (Ser536) in livers of HFD-fed and PBS-treated mice compared with LFD-fed controls; although this trend did not reach statistical significance. Upon treatment with metformin, the phosphorylation of liver JNK1 (p46) was significantly decreased compared with that of HFD-fed and PBS-treated mice (Figure 3B). When liver mRNA levels of proinflammatory cytokines were examined, HFD-fed and PBS-treated mice displayed a significant increase in the mRNA levels of interleukin-6 (IL-6), a proinflammatory cytokine that is abundantly expressed in hepatocytes; although HFD-fed and PBS-treated mice showed a decrease in liver mRNA levels of IL-1β and tumor necrosis factor α (TNFα) compared with LFD-fed and PBS-treated mice. Within all HFD-fed mice, treatment with metformin significantly decreased liver mRNA levels of IL-6, as well as IL-1β and TNFα compared with PBS (Figure 3C). Together, these results suggest that metformin decreases liver inflammatory responses while improving hepatic steatosis in DIO mice.

### Metformin treatment does not alter HFD-induced adiposity and adipose tissue inflammatory responses

**Figure 4:**
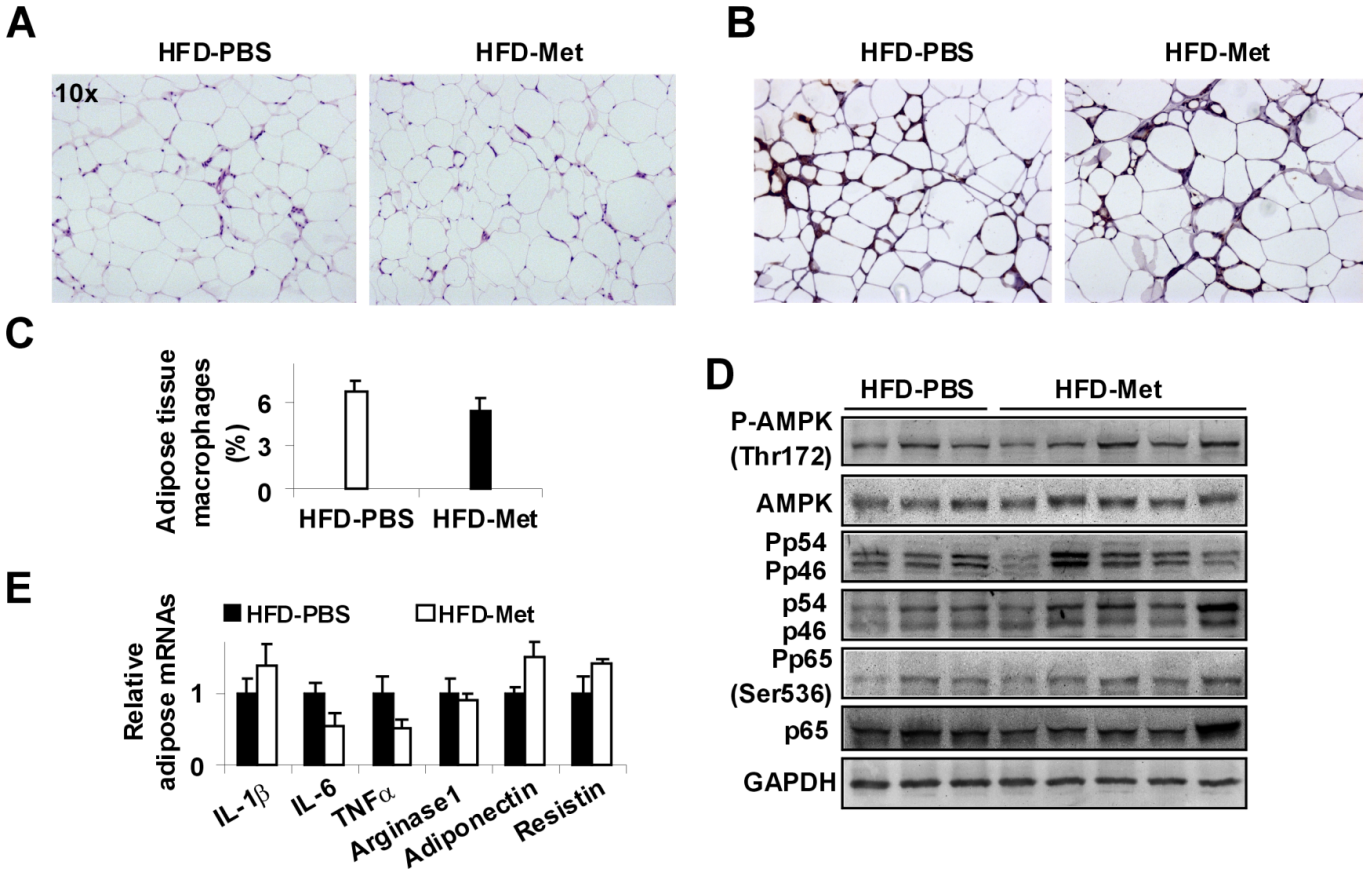
Metformin treatment does not alter HFD-induced adiposity and adipose tissue inflammation. Mice were treated as described in Figure 1. After the feeding/treatment regimen, mice were fasted for 4 hr prior to collection of tissue samples. (A) Adipose tissue histology (H&E staining). (B) Adipose tissue sections were stained for F4/80^+^ cells. (C) Adipose tissue macrophage infiltration. Percentages of mature macrophages (F4/80^+^ CD11b^+^ cells) in adipose tissue stromal cells were calculated using FACS analyses (n = 4–6). (D) Adipose tissue AMPK signaling and inflammatory signaling were examined using Western blot analyses (n = 4–6). (E) The mRNA levels of adipose genes were quantified using real-time PCR (n = 4–6). For bar graphs (C and E), data are means ± SE.

During obesity, dysfunctional adipose tissue critically contributes to the development of NAFLD [12]. We examined changes in adipose tissue phenotype. Consistent with DIO, all HFD-fed mice displayed a marked increase in visceral fat mass compared with LFD-fed mice (data not shown). Following treatment with metformin, HFD-fed mice did not display significant changes in visceral fat mass compared with HFD-fed and PBS-treated mice. Additionally, the size of adipocytes in HFD-fed and metformin-treated mice did not differ from that in HFD-fed and PBS-treated mice indicated by adipose tissue histology (Figure 4A). Thus, treatment with metformin did not alter HFD-induced adiposity. We next examined adipose tissue inflammatory responses. As indicated by percentages of mature macrophages (F4/80^+^ CD11b^+^ cells) from the isolated adipose tissue stromal vascular cells (Figure S1, A and B in File S1), adipose tissue macrophage infiltration in HFD-fed and PBS-treated mice was markedly increased compared with that in LFD-fed and PBS-treated mice (Figure S1C in File S1). However, within HFD-fed mice, treatment with metformin did not significantly alter adipose tissue macrophage infiltration (Figure 4C and Figure S1C in File S1). These results were consistent with the finding that adipose tissue content of F4/80^+^ cells in HFD-fed and metformin-treated mice did not differ from that in HFD-fed and PBS-treated mice (Figure 4B). Further analyses indicated that the percentages of proinflammatory macrophages (F4/80^+^ CD11b^+^ CD11c^+^ CD206^−^ cells) among mature adipose tissue macrophages in HFD-fed and metformin-treated mice also did not differ from those in HFD-fed and PBS-treated mice (data not shown). Unlike changes in liver AMPK signaling, adipose tissue AMPK signaling (phosphorylation) was not altered by metformin. Additionally, metformin treatment did not alter adipose tissue inflammatory signaling, indicated by the phosphorylation of JNK1 (p46) and NF-κB p65 (Ser536) (Figure 4D). When proinflammatory cytokine expression was examined for HFD-fed mice, treatment with metformin did not significantly alter adipose tissue mRNA levels of IL-1β, IL-6, and TNFα (Figure 4E), as well as mRNA levels of arginase 1, adiponectin, and resistin, which all are related to adipose tissue inflammation and function. Therefore, treatment with metformin for 4 weeks appears to have limited effects on altering adipose tissue phenotype in obese mice, indicated by adiposity and inflammatory responses.

### Metformin treatment inhibits hepatocyte fat deposition and inflammatory responses

**Figure 5:**
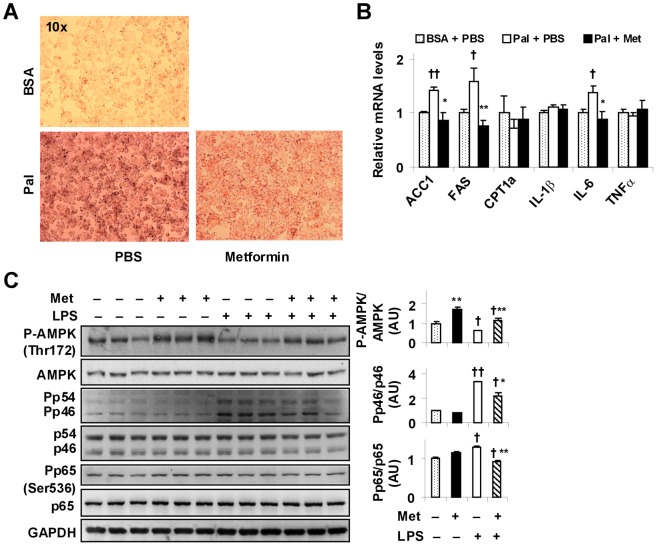
Metformin treatment blunts hepatocyte fat deposition, increases AMPK phosphorylation, and decreases inflammatory responses. (A) Hepatocyte fat deposition. Bottom panels, cells were incubated with palmitate (Pal). (B) Hepatocyte mRNA levels were quantified using real-time PCR. For A and B, H4IIE cells were treated with metformin (500 µM) or PBS in the presence of palmitate (250 µM) or bovine serum albumin (BSA) for 24 hr (B) and stained with Oil-Red-O for the last 1 hr (A). (C) Hepatocyte AMPK signaling and inflammatory signaling were examined using Western blot analyses. H4IIE cells were treated with metformin (500 µM) or PBS for 24 hr in the presence or absence of LPS (100 ng/ml) for the last 30 min. For bar graphs (B and C), data are means ± SE, n = 6–8. ^†^, *P*<0.05 and ^††^, *P*<0.01 Pal + PBS vs. BSA + PBS (B) or LPS vs. PBS (without LPS) under the same condition (with or without Met) (C); *, *P*<0.05 and **, *P*<0.01 Pal + Met vs. Pal + PBS (B) or Met vs. PBS (without Met) under the same condition (with or without LPS) (C).

As indicated by the results listed above (Figures 2, 3, 4), the liver is the primary target through which metformin ameliorates NAFLD in obese mice. We next examined the direct effects of metformin on metabolic and inflammatory responses in H4IIE cells, a rat hepatoma cell line commonly used as a cell model of NAFLD [28], [29]. In the absence of metformin, treatment of H4IIE cells with palmitate induced a significant increase in fat deposition (Figure 5A). This effect of palmitate, however, was partially blunted by metformin treatment, indicating a direct effect of metformin on inhibiting hepatocyte fat deposition. Consistent with changes in hepatocyte fat deposition, the mRNA levels of ACC1 and FAS in palmitate-treated H4IIE cells were significantly increased compared with those in control cells (Figure 5B). Following treatment with metformin, the stimulatory effect of palmitate on the expression of ACC1 and FAS was blunted (Figure 5B). In H4IIE cells, CPT1a mRNA levels were not significantly altered by palmitate in the absence or presence of metformin (Figure 5B). We also examined the mRNA levels of proinflammatory cytokines. Compared with the control, the mRNA levels of IL-6 were increased in palmitate-treated H4IIE cells. This stimulatory effect of palmitate was completely blunted by treatment with metformin (Figure 5B).

We examined AMPK signaling and inflammatory signaling in H4IIE cells. Compared with that in cells treated with control (in the absence of metformin and lipopolysaccharide (LPS)), the phosphorylation of AMPK was increased in metformin-treated cells (in the absence of LPS) (Figure 5C). In the presence of LPS alone, AMPK phosphorylation was decreased compared with that in control cells (in the absence of metformin and LPS), and this decrease was partially reversed by treatment with metformin. When inflammatory signaling was examined, metformin treatment did not significantly alter the phosphorylation of JNK1 (p46) and NF-κB p65 under the basal conditions (in the absence of LPS). However, metformin treatment significantly blunted the effect of LPS on increasing hepatocyte phosphorylation of JNK1 (p46) and NF-κB p65 compared with that in control (PBS)-treated hepatocytes. Together, these results suggest that metformin directly decreases hepatocyte fat deposition and suppresses hepatocyte inflammatory responses.

### Metformin treatment suppresses macrophage proinflammatory activation

**Figure 6:**
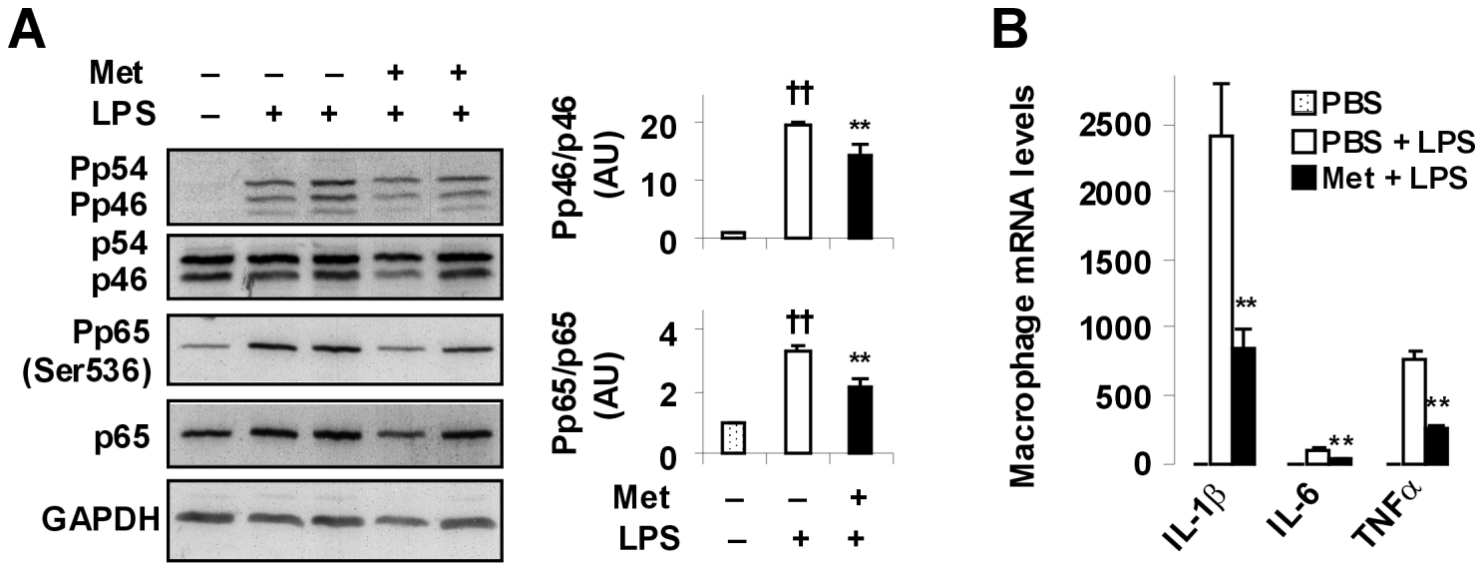
Metformin treatment suppresses macrophage proinflammatory activation. Bone marrow-derived macrophages were treated with metformin (500 µM) or PBS for 24 hr in the presence or absence of LPS (100 ng/ml) for the last 30 min (A) or 6 hr (B). (A) Macrophage inflammatory signaling was examined using Western blot analyses. (B) Macrophage mRNA levels of proinflammatory cytokines were quantified using real-time PCR. For bar graphs (A and B), data are means ± SE, n = 4–6. ^††^, *P*<0.01 LPS vs. PBS (without LPS) in the absence of metformin (A); **, *P*<0.01 Met vs. PBS (without metformin) in the presence of LPS (A) or Met + LPS vs. PBS + LPS (B).

Macrophages/Kupffer cells play a critical role in controlling the development of hepatic steatosis and inflammation [30]. We examined a direct effect of metformin on macrophage proinflammatory activation. In the absence of metformin, bone marrow-derived macrophages (BMDM) exhibited a significant increase in the phosphorylation of both JNK1 (p46) and NF-κB p65 (Ser536) in response to LPS stimulation (Figure 6A), indicating LPS induction of macrophage proinflammatory activation. However, upon treatment with metformin, the effect of LPS on inducing the phosphorylation of JNK1 (p46) and NF-κB p65 (Ser536) was partially blunted. When macrophage expression of proinflammatory cytokines was analyzed, the mRNA levels of IL-1β, IL-6, and TNFα were markedly increased in control (PBS)-treated BMDM after LPS stimulation. However, in metformin-treated macrophages, the effect of LPS on increasing proinflammatory cytokine mRNA levels was significantly lessened (Figure 6B). Together, these results suggest that metformin has a direct effect on inhibiting macrophage proinflammatory activation.

## Discussion

On an HFD, C57BL/6J mice developed obesity-associated insulin resistance, as well as hepatic steatosis and inflammation. Accordingly, HFD-fed mice were used as a model of NAFLD to assess the therapeutic effects of metformin. Consistent with the results obtained from both human subjects and rodent models [13], [23], [31], metformin treatment not only improved HFD-induced systemic insulin resistance and glucose intolerance, but also brought about a marked decrease in the severity of HFD-induced hepatic steatosis and inflammation. Using *in vitro* systems involving hepatocytes and macrophages, the present study demonstrates that metformin is capable of directly decreasing hepatocyte fat deposition and suppressing hepatocyte and macrophage inflammatory responses, which all are associated with increased AMPK phosphorylation. However, metformin treatment did not alter obesity-associated adipose tissue phenotype. Notably, HFD-induced adiposity and adipose tissue inflammation indicated by adipose tissue inflammatory signaling, macrophage infiltration, and cytokine expression in metformin-treated mice did not differ from those in control mice. Thus, the present study provides evidence to support that metformin treatment ameliorates obesity-associated hepatic steatosis and inflammation in a manner independent of adipose tissue phenotype; although obesity-associated adiposity and adipose tissue dysfunction are thought to critically contribute to the development of NAFLD.

Metformin is considered as an activator of AMPK. The latter, when in active form(s), exhibits an anti-lipogenic effect through suppressing hepatic expression of lipogenic enzymes including ACC1 and FAS [32]. In addition, active AMPK phosphorylates and inhibits ACC1/2 [33]. This leads to a decrease in the production of malonyl-CoA, which in turn releases the inhibitory effect on CPT1a to favor fatty acid oxidation. In combination, these effects of active AMPK are thought to account, to a large extent, for metformin actions on reducing hepatic steatosis. In the present study, two lines of evidence support that metformin acts through stimulating AMPK phosphorylation to reduce hepatic steatosis. Firstly, in the *in vivo* study, changes in liver AMPK phosphorylation, indicative of AMPK activity, were positively correlated with the phosphorylation of liver ACC and reversely correlated with the degree of hepatic steatosis and with changes in the mRNA levels of ACC1 and FAS. Secondly, using an *in vitro* system in which palmitate-treated hepatocytes served as a cell model of hepatic steatosis, the present study further supports the direct effects of metformin on suppressing hepatocyte fat deposition and on increasing hepatocyte AMPK phosphorylation. In terms of regulating hepatic steatosis, liver macrophages/Kupffer cells have been previously shown to critically control hepatocyte fat deposition [30]. Considering this, the *in vitro* results of the present study are significant in that a direct effect of metformin on suppressing hepatocyte fat deposition likely is sufficient enough to mediate the anti-hepatic steatosis actions of metformin.

The present study also suggests a potential link between AMPK and liver inflammation that is altered by metformin. In support of this, reversal of HFD-induced decrease in AMPK phosphorylation by metformin was accompanied by decreases in the phosphorylation of liver JNK1 (p46) and in the mRNA levels of proinflammatory cytokines. This observation was consistent with the results of a recent study in which metformin decreased liver inflammatory responses in mice fed both a methionine- and choline-deficient diet (MCD) and an HFD [23]. However, the study involving MCD/HFD-fed mice did not address how metformin suppresses liver inflammation, which could originate from hepatocyte fat deposition, macrophage/Kupffer cell proinflammatory activation, and/or adipose tissue inflammation [28]–[30], [34]. Because metformin did not alter HFD-induced adipose tissue phenotype, the present study examined the direct anti-inflammatory effects of metformin in both hepatocytes and macrophages. Of interest, treatment with metformin partially blunted the effect of LPS on inducing the phosphorylation of JNK1 (p46) and NF-κB p65 and on increasing the mRNA levels of proinflammatory cytokine(s) in cultured hepatocytes and macrophages. These results serve as convincing evidence to support that metformin acts directly on both hepatocytes and macrophages/Kupffer cells to suppress liver inflammation in obesity-associated NAFLD. It should be noted that metformin has a direct effect on reducing hepatocyte fat deposition as discussed above. This effect of metformin is believed to decrease hepatocyte production (release) of free fatty acids including palmitate, a potent proinflammatory fatty acid [28], [29]. Considering this, it is likely that in the *in vivo* condition metformin actions on hepatocytes also generate an indirect effect on macrophages/Kupffer cells (via paracrine) to suppress liver inflammation in NAFLD. In other words, metformin likely also acts through reducing hepatocyte fat deposition to indirectly suppress macrophage/Kupffer cell inflammatory activation. Future study is needed to examine the proportional contribution of hepatocytes vs. macrophages/Kupffer cells to the anti-inflammatory effects of metformin.

As illustrated by widely accepted concepts, dysfunctional adipose tissue during obesity contributes to the pathogenesis of NAFLD by increasing the delivery of fatty acids and inflammatory mediators to the liver to exacerbate hepatic fat deposition and inflammatory responses. Because of this, we also postulated that improved adipose tissue phenotype would contribute to the anti-NAFLD effect of metformin. However, this was not the case. In the present study, adipose tissue, unlike the liver, did not respond to metformin treatment. Notably, HFD-induced adiposity and adipose tissue inflammation in metformin-treated mice did not differ from those in PBS-treated control mice. The underlying mechanisms remained to be elucidated, but may be attributable to the effect that metformin treatment did not alter adipose tissue AMPK phosphorylation. Indeed, metformin actions on adipose tissue phenotype remain controversial. While metformin is shown to reduce body weight (adiposity) in both human and rodent models, a number of papers also demonstrate that metformin does not alter body weight, in particular in rodents fed an HFD [18], [35], [36]. Additionally, the weight-loss effect of metformin is tied closely to a decrease in food intake, leading to a question of whether or not metformin directly acts on adipose tissue. Considering this, the beneficial effects of metformin on features of NAFLD appear to be due largely to the direct effects of metformin on the liver. What should also be mentioned is that metformin is capable of countering insulin-induced suppression of muscle fatty acid oxidation and promoting triglyceride storage in skeletal muscle [37]. These effects may also contribute to the beneficial effects of metformin on features of NAFLD.

As mentioned above, metformin directly suppressed macrophage inflammatory activation *in vitro*. However, this was not the case for the *in vivo* study, in which metformin treatment did not alter the inflammatory status of adipose tissue macrophages. Given that metformin did not alter adipose tissue AMPK, it is possible that the treatment regimen used by the present study was not sufficient enough for metformin to be effective in adipose tissue. To verify this, future studies are required to include a relative long period of time and/or a higher dose for metformin treatment. Additional to adipose tissue inflammatory status, adiposity of HFD-fed mice was also not reduced by metformin in the present study. When adipose and liver tissues were compared, adipose tissue macrophages interacted with a highly proinflammatory local environment likely due to excessive fat deposition whereas liver macrophages/Kupffer cells did not. Considering this, it is also possible that a potential anti-inflammatory action of metformin on adipose tissue macrophages was offset by the proinflammatory effects of adipocyte-derived factors in particular saturated fatty acids [38]. While this point needs to be further validated, it appears to be clear that metformin primarily targets the liver to improve features of NAFLD, and this effect of metformin is independent of adipose tissue phenotype.

In summary, the present study provides evidence to support the beneficial effects of metformin on reducing hepatic steatosis and inflammation of NAFLD, and these effects are independent of alterations of adipose tissue phenotype. Mechanistically, metformin actions are attributable to the effects of metformin on improving hepatocyte fat metabolism and on suppressing hepatocyte and macrophage inflammatory responses. As such, metformin supplementation could be an effective approach for treatment and/or prevention of obesity-associated NFALD.

## Materials and Methods

### Animal experiments

C57BL/6J mice were obtained from the Jackson Laboratory and maintained on a 12:12-h light-dark cycle (lights on at 06:00). At 5–6 weeks of age, male mice were fed a high-fat diet (HFD, 60% fat calories, 20% protein calories, and 20% carbohydrate calories) for 12 weeks and treated with metformin (150 mg/kg body weight/d, solutions in PBS) or PBS via oral gavages for the last 4 weeks. As additional controls, gender- and age-matched C57BL/6J mice were fed a low-fat diet (LFD, 10% fat calories, 20% protein calories, and 70% carbohydrate calories) for 12 weeks and treated with PBS for the last 4 weeks. Both diets are products of Research Diets, Inc (New Brunswick, NJ) and contain the same of amount of casein, L-cystein, cellulose, sucrose, soybean oil, and minerals. However, the HFD contains much more lard but none corn starch compared with the LFD. During the 12-week feeding/treatment period, body weight and food intake of the mice were recorded weekly. After the feeding/treatment regimen, mice were fasted for 4 hr before sacrifice for collection of blood and tissue samples [39]–[41]. Epididymal, mesenteric, and perinephric fat depots were dissected and weighed as visceral fat content [40]. Liver weight was also recorded. After weighing, part of epididymal fat was subjected to isolation of stromal vascular cells as described below. Additional adipose and liver tissue samples were either fixed and embedded for histological analyses (H&E staining) or frozen in liquid nitrogen and stored at −80°C for further analyses [40], [42]. Some mice were fasted similarly and used for insulin and glucose tolerance tests as described below. All animals received human care and all study protocols were approved by the Institutional Animal Care and Use Committee of Texas A&M University.

### Insulin and glucose tolerance tests

Mice were fasted for 4 hr and received an intraperitoneal injection of insulin (1 U/kg body weight) or D-glucose (2 g/kg body weight). For insulin tolerance tests, blood samples (5 µl) were collected from the tail vein before and at 15, 30, 45, and 60 min after the bolus insulin injection. Similarly, for glucose tolerance tests, blood samples were collected from the tail vein before and at 30, 60, 90 and 120 min after the glucose bolus injection [43], [44]. The levels of plasma glucose were measured using an enzymatic assay kit (Sigma, St. Louis, MO).

### Isolation of stromal vascular cells from adipose tissue

Adipose tissue stromal vascular cells (SVC) were isolated using the collagenase digestion method as previously described [43], [45]. After digestion and centrifugation, the pelleted cells were collected as SVC and subjected to FACS analyses.

### Flow cytometry analysis

Adipose tissue SVC were stained with fluorescence-tagged antibodies: anti-F4/80, anti-CD11b for macrophages, and anti-CD11c and anti-CD206 for macrophage inflammatory status as previously described [46], and subjected to FACS analyses using BD FACSAria II flow cytometer (BD Biosciences, San Jose, California, USA) that is operated by Texas A&M Health Science Center College of Medicine Cell Analysis Facility.

### Oil-Red-O staining and immunohistochemical analyses

Frozen liver sections were stained with Oil-Red-O as previously described [32]. The paraffin-embedded liver and adipose tissue blocks were cut into sections of 5 µm thickness and stained for the expression of F4/80 with rabbit anti-F4/80 (1∶100) (AbD Serotec, Raleigh, NC) [43].

### Cell culture and treatment

H4IIE cells (rat hepatoma cells) were maintained in high glucose Dulbecco's modified eagle medium (DMEM) supplemented with 10% fetal bovine serum, 100 units/ml penicillin and 100 µg/l streptomycin as previously described [28], [34]. At 80% confluence, H4IIE cells were treated with metformin (500 µM) or PBS in the presence or absence of palmitate (250 µM) for 24 hr to induce fat deposition. To examine lipid accumulation, the treated hepatocytes were stained with Oil-Red-O for the last 1 hr. To determine changes in hepatocyte AMPK and inflammatory signaling, metformin- or PBS-treated cells were supplemented with or without LPS (100 ng/ml) for 30 min prior to harvest. Cell lysates were prepared and used to measure the levels of AMPK, JNK1, NF-κB p65, phospho-AMPK, phospho-JNK1, and phospho-p65 (Ser536) using Western blot analyses. To analyze hepatocyte gene expression, the total RNA was prepared from metformin- or PBS-treated cells and subjected to reverse transcription and real-time PCR.

Additional to hepatocytes, macrophages were also used to address a direct anti-inflammatory effect of metformin. Briefly, bone marrow cells were isolated from the tibias and femurs of chow diet-fed C57BL/6J mice as previously described [47]. After differentiation with Iscove's modified Dulbecco's medium (IMDM) containing 10% fetal bovine serum and 15% L929 culture supernatant for 8 days, bone marrow-derived macrophages (BMDM) were treated with metformin (500 µM) or PBS for 24 hr in the presence or absence of LPS (100 ng/ml) for the last 30 min. Cell lysates were prepared and used to examine the inflammatory signaling using Western blot analyses. Some cells were treated with or without LPS at the same dose for 6 hr prior to harvest of RNA samples.

### Western blots

Lysates were prepared from frozen tissue samples and cultured cells using the lysis buffer containing 50 mm HEPES (pH 7.4), 1% Triton X-100, 50 mm sodium pyrophosphate, 0.1 m sodium fluoride, 10 mm EDTA, 10 mm sodium orthovanadate, 10 µg/ml aprotinin, 10 µg/ml leupeptin, 2 mm benzamidine, and 2 mm phenylmethylsulfonyl fluoride. After protein electrophoresis and transfer, immunoblots were performed using rabbit anti-serum as primary antibody at a 1∶1,000 dilution. The blot was followed by a 1∶10,000 dilution of goat anti-rabbit horseradish peroxidase-conjugated secondary antibody kit (Immobilon™ Western; EMD Millipore, Billerica, MA, USA) as previously described [40]. GAPDH was used as a loading control. The maximum intensity of each band was quantified using ImageJ software. Ratios of P-AMPK/AMPK, P-ACC/ACC, Pp46/p46, and Pp65/p65 were normalized to GAPDH and adjusted relative to the average of PBS-treated control, which was arbitrarily set as 1 (AU).

### RNA isolation, reverse transcription, and real-time PCR

The total RNA was isolated from frozen tissue samples and cultured/isolated cells. Reverse transcription was performed using the GoScript™ Reverse Transcription System (Promega) and real-time PCR analysis was performed using SYBR Green (LightCycler® 480 system; Roche) [28], [41], [48]. The mRNA levels were analyzed for ACC1, FAS, CPT1a, SREPB1c, IL-1β, IL-6, TNFα, arginase 1, adiponectin, and/or resistin in tissue and/or cell samples. A total of 0.1 µg RNA was used for the determination. Results were normalized to 18 s ribosomal RNA as plotted as relative expression to the average of PBS-treated control, which was set as 1.

### Statistical Methods

Numeric data are presented as means ± SE (standard error). Two-tailed Student's *t* tests were used for statistical analyses. Differences were considered significant at the *P*<0.05.

## Supporting Information

File S1 **Contains Figure S1. FACS analysis of adipose tissue stromal vascular cells.** Male C57BL6/J mice, at 5–6 weeks of age, were fed a high-fat diet (HFD) and treated with metformin (Met, 150 mg/kg/d, in phosphate-buffered saline (PBS)) or PBS for the last 4 weeks of HFD feeding (n = 4–6). After the feeding/treatment regimen, stromal vascular cells (SVC) were isolated from epididymal fat pads and subjected to FACS analysis. (A) SVC were included for FACS analyses. (B) SVC (without staining) were analyzed for APC and FITC. (C) SVC (with staining) were analyzed for F4/80 (FITC) and CD11b (APC) expression.
(PDF)
