# Supplementary material for: Metformin Ameliorates Hepatic Steatosis and Inflammation without Altering Adipose Phenotype in Diet-Induced Obesity
Source: PLoS One. 2014 Mar 17;9(3):e91111. doi: 10.1371/journal.pone.0091111 (PMC3956460; doi:10.1371/journal.pone.0091111)
Supplement: File S1 — Contains Figure S1. FACS analysis of adipose tissue stromal vascular cells. Male C57BL6/J mice, at 5–6 weeks of age, were fed a high-fat diet (HFD) and treated with metformin (Met, 150 mg/kg/d, in phosphate-buffered saline (PBS)) or PBS for the last 4 weeks of HFD feeding (n = 4–6). After the feeding/treatment regimen, stromal vascular cells (SVC) were isolated from epididymal fat pads and subjected to FACS analysis. (A) SVC were included for FACS analyses. (B) SVC (without staining) were analyzed for APC and FITC. (C) SVC (with staining) were analyzed for F4/80 (FITC) and CD11b (APC) expression. (PDF) [file pone.0091111.s001.pdf]

Supporting information

Supplemental Figure S1

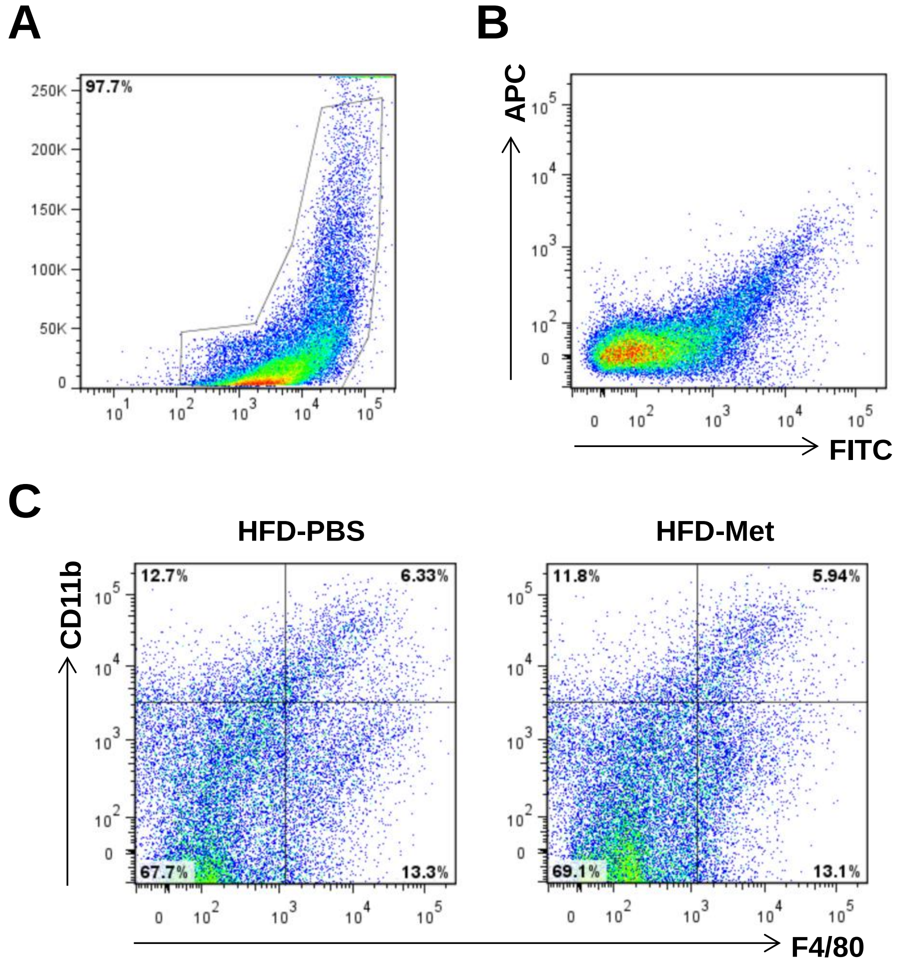

Figure S1 legend

Figure S1 FACS analysis of adipose tissue stromal vascular cells

Male C57BL6/J mice, at 5 – 6 weeks of age, were fed a high-fat diet (HFD) and treated with metformin (Met, 150 mg/kg/d, in phosphate-buffered saline (PBS)) or PBS for the last 4 weeks of HFD feeding (n = 4 – 6). After the feeding/treatment regimen, stromal vascular cells (SVC)

were isolated from epididymal fat pads and subjected to FACS analysis. (A) SVC were included for FACS analyses. (B) SVC (without staining) were analyzed for APC and FITC. (C) SVC (with staining) were analyzed for F4/80 (FITC) and CD11b (APC) expression.
